# 772 Analysis of Therapeutic Time and Functional Outcomes After a 92% TBSA Burn Injury

**DOI:** 10.1093/jbcr/irae036.313

**Published:** 2024-04-17

**Authors:** Maggie McCool, McKenzie Stevens, Laura Stephens, Kevin Johnson, Nathan Heppermann, Nicole P Bernal

**Affiliations:** Ohio State University Wexner Medical Center, Columbus, OH; The Ohio State University, Columbus, OH; The Ohio State University Wexner Medical Center, Galena, OH; The Ohio State University Wexner Medical Center, Hilliard, OH; the ohio state university hospital wexner medical center, Columbus, OH; The Ohio State University Wexner Medical Center, Columbus, OH; Ohio State University Wexner Medical Center, Columbus, OH; The Ohio State University, Columbus, OH; The Ohio State University Wexner Medical Center, Galena, OH; The Ohio State University Wexner Medical Center, Hilliard, OH; the ohio state university hospital wexner medical center, Columbus, OH; The Ohio State University Wexner Medical Center, Columbus, OH; Ohio State University Wexner Medical Center, Columbus, OH; The Ohio State University, Columbus, OH; The Ohio State University Wexner Medical Center, Galena, OH; The Ohio State University Wexner Medical Center, Hilliard, OH; the ohio state university hospital wexner medical center, Columbus, OH; The Ohio State University Wexner Medical Center, Columbus, OH; Ohio State University Wexner Medical Center, Columbus, OH; The Ohio State University, Columbus, OH; The Ohio State University Wexner Medical Center, Galena, OH; The Ohio State University Wexner Medical Center, Hilliard, OH; the ohio state university hospital wexner medical center, Columbus, OH; The Ohio State University Wexner Medical Center, Columbus, OH; Ohio State University Wexner Medical Center, Columbus, OH; The Ohio State University, Columbus, OH; The Ohio State University Wexner Medical Center, Galena, OH; The Ohio State University Wexner Medical Center, Hilliard, OH; the ohio state university hospital wexner medical center, Columbus, OH; The Ohio State University Wexner Medical Center, Columbus, OH; Ohio State University Wexner Medical Center, Columbus, OH; The Ohio State University, Columbus, OH; The Ohio State University Wexner Medical Center, Galena, OH; The Ohio State University Wexner Medical Center, Hilliard, OH; the ohio state university hospital wexner medical center, Columbus, OH; The Ohio State University Wexner Medical Center, Columbus, OH

## Abstract

**Introduction:**

Patients who present with limited donor site availability often require increased time for wound closure due to limited grafting options. Large total body surface area (TBSA) burns have a higher risk for significant scarring, infections and deconditioning. Patients with a large TBSA present unique challenges in participating consistently with therapy services due to the number of surgical interventions with post-operative restrictions. We hypothesized that frequent surgical interventions with restrictions would negatively impact Physical Therapy (PT) and Occupational Therapy (OT) minutes and Activity Measure for Post-Acute Care (AM-PAC) scores.

Case details: A 32-year-old male sustained a 92% TBSA injury due to an accelerant flame burn with an expected mortality rate of 78%. Over 70% of his body experienced full thickness burns. He was admitted to the hospital for 364 days achieving 95% wound closure at time of discharge.

**Methods:**

This case study looked at the following factors: surgical intervention with post-op restrictions, total PT/OT minutes spent with the patient, and functional performance using AM-PAC scores. The patient/family signed consent for use of his information.

Data was acquired by a retrospective analysis from the electronic medical record and included daily PT/OT minutes and AM-PAC scores.

**Results:**

The patient required 30 surgical interventions, 10 of which included prolonged post-op restrictions; 3 due to the use of cultured endothelial cells. He received a total of 24,940 minutes from PT/OT over 291 days.

During the period of surgical interventions, PT/OT AM-PAC scores remained low, varying between 6 and 8, out of 24. The patient experienced incremental improvements in the AM-PAC followed by quick regressions. A total of 10 PT/3 OT regressions continued until completion of large-scale autografting surgeries. After this time, a consistent rise in AM-PAC was achieved. At discharge, the PT AM-PAC score was 17 and the OT AM-PAC score was 13.

The relationship between total PT/OT minutes over time remained highly variable with few trends noted.

**Conclusions:**

We found that frequent surgical interventions with post-op restrictions suppressed improvements in AM-PAC scores without impacting total therapy time. The rise in AM-PAC scores may correlate to an increase in medical stability and transfer out of the intensive care unit, which occurred around the time of his last surgery. PT/OT minutes over time were not impacted, likely due to the number of available interventions for a patient with a large TBSA burn. Limitations in using the AM-PAC in the burn population include decreased ability to capture ROM improvements, compensatory strategies and activity tolerance.

**Applicability of Research to Practice:**

Large TBSA burns provide a unique challenge to burn units and successful outcomes offer an opportunity to improve our practice. Time based studies linked to functional outcomes are imperative to guide allocation and staffing of therapy services.

